# Genotypic variation in a foundation tree (*Populus tremula* L.) explains community structure of associated epiphytes

**DOI:** 10.1098/rsbl.2014.0190

**Published:** 2014-04

**Authors:** Chantel Davies, Christopher J. Ellis, Glenn R. Iason, Richard A. Ennos

**Affiliations:** 1 Institute of Evolutionary Biology, University of Edinburgh, Mayfield Road, EH9 3JT, UK; 2 Royal Botanic Garden Edinburgh, 20A Inverleith Row, Edinburgh EH3 5LR, UK; 3 The James Hutton Institute, Craigiebuckler, Aberdeen AB15 8QH, UK

**Keywords:** aspen clone, community genetics, environment, epiphyte, randomized trial

## Abstract

Community genetics hypothesizes that within a foundation species, the genotype of an individual significantly influences the assemblage of dependent organisms. To assess whether these intra-specific genetic effects are ecologically important, it is required to compare their impact on dependent organisms with that attributable to environmental variation experienced over relevant spatial scales. We assessed bark epiphytes on 27 aspen (*Populus tremula* L.) genotypes grown in a randomized experimental array at two contrasting sites spanning the environmental conditions from which the aspen genotypes were collected. We found that variation in aspen genotype significantly influenced bark epiphyte community composition, and to the same degree as environmental variation between the test sites. We conclude that maintaining genotypic diversity of foundation species may be crucial for conservation of associated biodiversity.

## 1. Introduction

Foundation species are key-stone elements in ecosystems [1] whose genetically determined character variation may structure associated communities [2]. Studies have demonstrated an effect of foundation species genotype on the composition of associated communities within hybrid zones, a situation characterized by segregation of large interspecific genetic differences among individuals of the hybridizing species [3,4]. An increasing number of reports also document an effect of intra-specific genetic variation within foundation species on communities of associated taxa [5–7]. If widely confirmed, this intra-specific genetic effect would become a critically important consideration during ecosystem restoration, because the genetic variability within founder populations used to create habitat structure could significantly affect the accumulation of species diversity within dependent guilds.

To demonstrate the ecological relevance of intra-specific genetic variation, experimental studies must not only detect a significant effect on associated communities, but establish that the magnitude of this effect is comparable to that caused by environmental variation at a geographical scale equivalent to the sampling of genotypes [8]. These criteria can be met by establishing trials in which replicated genotypes of a foundation species are randomized in space at a number of different sites covering the range of environmental conditions from which they were sampled [9,10]. Communities of dependent species associating themselves with replicated genotypes across these sites could then be analysed to robustly estimate the effects of both foundation species genotype and environmental contrasts.

Here, we use a tree species which is readily cloned (European aspen, *Populus tremula* L.) to establish randomized and replicated trials of naturally occurring genotypes at two sites with strongly contrasting climatic characteristics. Aspen has known high levels of associated diversity, including conservation priority species which are specialists [11]. We assessed community composition of associated epiphytic lichens and bryophytes established after 15 years on clonal replicates, testing the relative importance of an environmental (site) effect, an intra-specific genetic effect and their interaction on epiphyte community composition.

## 2. Material and methods

### (a) Clonal trials

**Figure 1.**
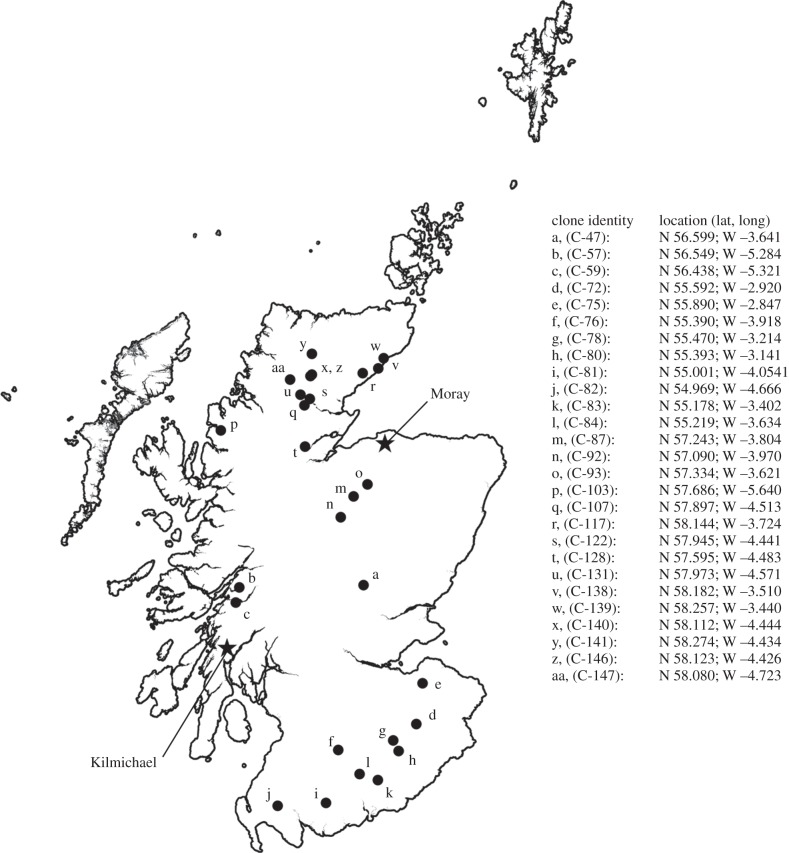
Site locations from which genetically different aspen clones were collected in Scotland (closed circles), and the position of the two contrasting experimental sites (stars) in which they were grown in randomized trials (Moray and Kilmichael, see the electronic supplementary material, table S1).

A root cutting from a single genotype (clone) of *P. tremula* L. was collected from each of 27 widely separated locations across Scotland [12,13] (figure 1). Previous work has indicated that aspen genetic diversity within Scotland is comparable to that elsewhere in the species’ range, and aspen clones collected from different locations within Scotland represent different genetic individuals [12]. Replicate cuttings from aspen clones were planted in randomized-block trials established in 1993/1994 [13], at two contrasting experimental sites in Scotland; at Kilmichael (latitude 56°06′22″ N, longitude 05°24′15″ W) and Moray (latitude 57°38′18″ N, longitude 03°23′48″ W). These sites represent the outer envelope of environmental variability characterized by a strong east–west climatic gradient (figure 1; electronic supplementary material, table S1). Four ramets of each clone were planted at 3 m intervals, in each of four or five randomized blocks for Kilmichael and Moray, respectively. Established trees were grown for 15 years, and the single most vigorous ramet of each clone from each block was assessed for epiphytes over the winter of 2009/2010.

### (b) Assessment of epiphyte communities

An established standard method [14] was used to quantify epiphyte community composition as frequency of occurrence in a 5 cm × 25 cm quadrat divided into sub-quadrats of 5 cm × 5 cm, each with 1 cm × 1 cm subunits. Species presence–absence was scored within quadrat subunits. Sampling was at breast height (130 cm) for cardinal points on the bole (N, S, E and W). Where species could not be identified in the field, they were examined at the Royal Botanic Garden Edinburgh using chemical spot tests, comparison with herbarium specimens and identification using high-power light microscopy.

### (c) Factors influencing epiphyte communities

Ordination of the epiphyte community composition for sampled ramets was performed using detrended correspondence analysis (DCA) [15]. Frequency cover values rescaled between 0 and 1 were square-root transformed, with rare species down-weighted. Ordination axes one and two were treated as community response variables, with species turnover along DCA axes partitioned into the unique effects of site and aspen genotype (within site) using analysis of variance, in addition to their G × E interaction. Genetically determined differences in epiphyte composition were examined by grouping the DCA scores for individual ramets according to clone identity, and comparing the means ± 1 s.e. among the different clones.

## 3. Results

**Figure 2.**
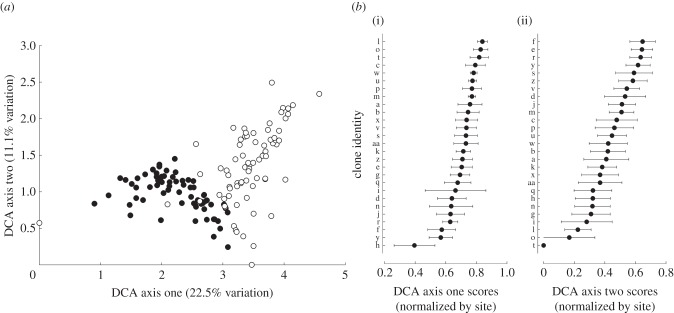
(*a*) Ordination of samples by DCA to determine epiphyte community variation among individual aspen ramets, coded by site identity (closed symbols, Moray; open symbols, Kilmichael). Note that some samples from the same site and with similar communities plot over one another. (*b*) Mean and 1 s.e. of sample scores normalized by site, and grouped by clone identity, plotted for (i) DCA axis one and (ii) DCA axis two, to demonstrate the variability in community composition among clones (for coding of aspen clones, see figure 1).

A total of 26 epiphytic taxa (23 lichen species, two mosses and one liverwort) were recorded on the aspen ramets assessed at the two sites (electronic supplementary material, table S2). DCA axes one and two explained 22.5% and 11.1%, respectively, of the variation in epiphyte community composition among ramets, with environmentally determined differences between the two sites clearly evident (figure 2*a*). Epiphyte community composition also varied among the aspen genotypes, whose mean DCA scores along both axes one and two are illustrated in figure 2*b*.

For DCA axes one and two, ANOVA showed significant effects (*p* < 0.05) of both aspen genotype and experimental site (table 1). For DCA axis one, the effect of site explained more variation than did aspen genotype, though with a significant interaction term. Conversely, aspen genotype uniquely explained the greater variation in epiphyte community composition along DCA axis two, with no significant interaction.

**Table 1.** Results of analysis of variance, to partition variation in epiphyte community composition along DCA axes one and two (figure 2) according to the main effects of site identity and aspen genotype, and their G × E interaction.

community response	main effects						interaction		
	site			genotype			site × genotype		
	*F*_1,142_	*p*	*R*^2^ (%)	*F*_27,142_	*p*	*R*^2^ (%)	*F*_27,142_	*p*	*R*^2^ (%)
DCA axis one	116.52	<0.001	33.5	1.57	0.049	12.2	1.74	0.021	13.5
DCA axis two	14.26	<0.001	5.61	2.12	0.003	22.5	1.5	0.067	15.9

Comparisons of individual species scores (electronic supplementary material, figure S1) clarified environmental effects on the distribution of taxa: lichen species adapted to a more continental climate, such as *Lecanora populicola* [16], had optima scores less than 2 on DCA axis one and were associated with the Moray aspen samples. By contrast, moisture-demanding mosses (*Hypnum* and *Orthotrichum* spp.) and liverworts (*Frullania dilatata*) had optima more than 4 and were associated with aspen ramets from the oceanic Kilmichael site.

## 4. Discussion

The experimental design used here allowed us to directly compare the amount of variation in epiphyte community composition explained by intra-specific genetic effects sampled over corresponding environmental space. Previous attempts to demonstrate that genetic variation of foundation species determines the community composition of associated species have been criticized on the grounds that genetic variants have been sampled over a large geographical area, and the differences between them have been tested in a single site [8–10]. Environmental differences within a site will be small relative to those between sites from which the genotypes have been sampled, serving to exaggerate the relative effects of genetic variation. In the present experiment, the sizes of genetic and environmental effects on associated community composition are directly comparable.

It is important that our two contrasting experimental sites approximate the outer bounds in a bioclimatic envelope from the hyper-oceanic west of Scotland to the relatively more continental northeast (figure 1) and are also different in terms of soil type. Previous studies have shown that naturally occurring aspen epiphyte communities are highly variable and functionally contrasting along this same bioclimatic gradient [17]. Nevertheless, we find that intra-specific genetic variation within aspen can have an importance that is comparable to the role of environment in structuring epiphyte communities. This includes an interaction effect in which the community response to foundation species genotype is dependent on environmental setting, as found in previous studies [18].

The magnitude of the host genotype effect on associated epiphyte community composition has potentially widespread implications for conservation. Forest stands with mixed aspen genotypes may generate higher levels of accumulated diversity because of contrasting species composition among clones. Our findings also indicate that a reduction in genetic diversity of a foundation species such as aspen is likely to lead to a decline in the diversity of the associated epiphyte communities, as suggested by studies conducted in natural systems over small scales [6]. This will have knock on effects for other forest biodiversity and ecosystem functions including nutrient capture and cycling, and food-web dynamics [19,20]. Such problems may be especially pertinent to forest restoration programmes where genetic diversity can be lost very rapidly [21]. This is particularly true for aspen in Scotland, where sexual reproduction is rare, and production of forest material takes place largely through propagation of root cuttings [22]. In this situation, it is essential to maintain a diverse mixture of clones for planting not only to allow the population of aspen to respond to future environmental change, but to ensure that the regenerated population is capable of supporting a diverse epiphytic flora.

## Data accessibility

Data are deposited in the Dryad database doi:10.5061/dryad.5c88j.
